# Post-discharge light physical activity indicates recovery in acutely hospitalized older adults – the Hospital-ADL study

**DOI:** 10.1186/s12877-023-04031-9

**Published:** 2023-05-19

**Authors:** Michel Terbraak, Daisy Kolk, Janet L. MacNeil Vroomen, Jos W. R. Twisk, Bianca M. Buurman, Marike van der Schaaf

**Affiliations:** 1 Center of Expertise Urban Vitality, Faculty of Health, Amsterdam University of Applied Sciences, Amsterdam, Netherlands; 2 Amsterdam UMC, Location University of Amsterdam, Cardiology, Meibergdreef 9, Amsterdam, Netherlands; 3 Amsterdam Cardiovascular Sciences, Atherosclerosis & Ischemic Syndromes, Amsterdam, Netherlands; 4 Department of Physical Therapy, Amsterdam University of Applied Sciences, Tafelbergweg 51, Amsterdam, 1105 BD Netherlands; 5 Amsterdam UMC, Location University of Amsterdam, Internal Medicine, Section of Geriatric Medicine, Amsterdam Public Health Research Institute, Meibergdreef 9, Amsterdam, Netherlands; 6 Department of Medicine for Older People, Amsterdam UMC, Location Vrije Universiteit Amsterdam, Boelelaan 1117, Amsterdam, Netherlands; 7 Epidemiology and Biostatistics, Amsterdam UMC, Location Vrije Universiteit Amsterdam, Boelelaan 1117, Amsterdam, Netherlands; 8 Amsterdam UMC, Location University of Amsterdam, Rehabilitation, Meibergdreef 9, Amsterdam, Netherlands; 9 Amsterdam Movement Sciences, Ageing and Vitality, Amsterdam, Netherlands

**Keywords:** Accelerometer, Physical performance, Rehabilitation, Post-acute care, Older patients, Frailty

## Abstract

**Background:**

Physical activity (PA) levels might be a simple overall physical function indicator of recovery in acutely hospitalized older adults; however it is unknown which amount and level of PA is associated with recovery. Our objective was to evaluate the amount and level of post discharge PA and its optimum cut-off values associated with recovery among acutely hospitalized older adults and stratified for frailty.

**Methods:**

We performed a prospective observational cohort study including acutely hospitalized older adults (≥ 70 years). Frailty was assessed using Fried’s criteria. PA was assessed using Fitbit up to one week post discharge and quantified in steps and minutes light, moderate or higher intensity. The primary outcome was recovery at 3-months post discharge. ROC-curve analyses were used to determine cut-off values and area under the curve (AUC), and logistic regression analyses to calculate odds ratios (ORs).

**Results:**

The analytic sample included 174 participants with a mean (standard deviation) age of 79.2 (6.7) years of whom 84/174 (48%) were frail. At 3-months, 109/174 participants (63%) had recovered of whom 48 were frail. In all participants, determined cut-off values were 1369 steps/day (OR: 2.7, 95% confidence interval [CI]: 1.3–5.9, AUC 0.7) and 76 min/day of light intensity PA (OR: 3.9, 95% CI: 1.8–8.5, AUC 0.73). In frail participants, cut-off values were 1043 steps/day (OR: 5.0, 95% CI: 1.7–14.8, AUC 0.72) and 72 min/day of light intensity PA (OR: 7.2, 95% CI: 2.2–23.1, AUC 0,74). Determined cut-off values were not significantly associated with recovery in non-frail participants.

**Conclusions:**

Post-discharge PA cut-offs indicate the odds of recovery in older adults, especially in frail individuals, however are not equipped for use as a diagnostic test in daily practice. This is a first step in providing a direction for setting rehabilitation goals in older adults after hospitalization.

**Supplementary Information:**

The online version contains supplementary material available at 10.1186/s12877-023-04031-9.

## Background

Minimum levels of physical activity (PA), defined as any bodily movement produced by skeletal muscles that requires energy expenditure such as household or ambulatory activities, are highly recommended to reduce disease and disability and decrease all-cause morbidity and mortality in older adults [1, 2]. Older adults are at increased risk on insufficient recovery after acute illness and hospitalization. This might be due to a lack of their physical resilience: the ability to resist or recover from functional decline after an acute health stressor [3]. Physical resilience is multifactorial and a dynamic process in response to health stressors such as acute illness and hospitalization. A loss in physical functioning can result in permanent functional decline, but also in readmission or mortality. Therefore, we defined recovery as prevention of physical decline, hospital readmission or mortality. PA has been identified as a physical function indicator of health [4] and as an indicator of post-discharge recovery in acutely hospitalized older adults [5]. Moreover, PA tracking is a non-invasive measurement that can be easily done during and after hospitalization. PA measurements may provide clinicians an easy tool for assessment of recovery in their patients, which is needed to make clinical decisions and to support functional rehabilitation. However, the association between post discharge PA levels and older adults recovery after hospitalization is unknown, and recommendations for PA levels for this population are lacking. If we know how much and at what intensity older people are physically active post discharge and which levels are associated with recovery, this may be a good first step to set rehabilitation goals.

Preferably, PA is objectively measured, e.g., with an accelerometer because activity trackers show greater construct validity than older adult’s generally overestimated self-reported PA [6, 7]. PA thresholds are often expressed in minutes of PA at specific levels of intensity or in daily step counts. The World Health Organization (WHO) [8] and PA Guidelines for Americans, 2^nd^ edition [9] recommend a minimum of 150–300 min of moderate PA every week for healthy adults. Together with normal daily activities, this translates to 7000–8000 steps per day [10]. This recommendation also applies to older adults living with chronic conditions or disability; however, it is unknown whether it applies to older adults who have recently been discharged from hospital [11].

Older adults typically take few steps after acute hospitalization; a median of 2000 steps per day in the first week after discharge has been reported [12]. After acute hospitalization, older adults are at high risk for adverse outcomes such as functional decline or hospital readmission [13, 14] particularly if they are frail [15]. Frailty is highly prevalent among acutely hospitalized older adults [16], and is characterized by reduced physical performance and PA, and a greater vulnerability to adverse outcomes [17].

Previous research found that older adults who took fewer than 900 steps per day during hospitalization were more likely to experience functional decline at discharge [18]. The number of steps taken in the first week post discharge has been associated with functional decline and readmission risk [12, 19], and may be an important underutilized PA physical function indicator of overall health and risk of readmission in older patients [19–22]. In addition to step counts, insight into PA intensity levels is important, as moderate to vigorous PA increases caloric expenditure and improves muscle mass and endothelial function [23]. Overall, a norm for the post-discharge amount and intensity of PA that differentiates patients who recover from those who do not is lacking.

As post-discharge PA might be a good physical function indicator of overall health, physical activity norms may be a first step to help clinicians to identify older adults at risk of insufficient recovery and may direct older adults towards recovery [17]. The aim of this study was to investigate the association of number of steps and minutes PA with recovery three months post-discharge for both continuous values, and optimum cut-off values of steps and PA-minutes that differentiate acutely hospitalized older adults who recover from those who do not. Secondly, we aimed to perform these analyses also stratified for frail and non-frail patients as we hypothesized that PA is a better indicator for recovery in frail than in non-frail patients.

## Methods

### Study participants

We included participants from the Hospital-Associated Disability and impact on daily Life (Hospital-ADL) study. This multicenter observational prospective cohort study investigated hospital-associated functional decline among adults aged 70 years and over, who were acutely admitted to Dutch hospitals for ≥ 48 h between October 2015 and June 2017 [24]. Participants were recruited from internal medicine, cardiology, and geriatric wards. Further inclusion criteria for the Hospital-ADL study were: 1] approval of the medical doctor; 2] Mini-Mental State Examination score ≥ 15; and 3] sufficient understanding of Dutch. Exclusion criteria were 1] a life expectancy of less than 3 months; or 2] need for help with all six basic activities of daily living (ADLs) (bathing, dressing, eating, toileting, transferring, and maintaining continence) [25]. For the present study, all participants of the Hospital-ADL study were asked to wear an activity tracker during and after hospital stay and were included after written informed consent was obtained.

The study was approved by the Institutional Review board of the Amsterdam University Medical Centers (A-UMC), Academic Medical Center in The Netherlands (Protocol ID: AMC2015_150).

This study was carried out according to the Dutch Medical Research Involving Human Subjects Act and principles of the Declaration of Helsinki (1964). Local approval was provided by all participating hospitals.

### Assessments

Trained researchers collected measurements according to standardized operating procedures. Baseline variables, including age, education, Charlson Comorbidity Index [26], polypharmacy, physical performance (Short Physical Performance Battery) [27], Functional Ambulation Categories (FAC) [28], and cognition (Mini-Mental State Examination) [29] were measured at inclusion (< 48 h after hospital admission).

#### Counting steps and measuring activity intensity

Physical activity has been identified as a physical function indicator of health [4], and as an indicator of recovery [5]. Preferably, PA is objectively measured, e.g., with an accelerometer. PA thresholds are often expressed in steps or minutes at a specific level of intensity. Therefore, we chose to investigate both steps and minutes of PA at specific levels of intensity.

We used the wrist worn Fitbit Flex activity tracker (Fitbit, Inc., San Francisco) to count steps and minutes of PA at different intensities. The Fitbit is user-friendly with a low risk of participant withdrawal, and tracks PA equally accurate as the gold standard Actigraph (*r* = 0.96) in healthy adults [30, 31] and older adults [32, 33], although steps may be underestimated and more variation (up to 30%) is introduced at reduced walking speeds or lower PA levels [32, 33]. Participants were instructed to wear the Fitbit continuously on the non-dominant wrist for seven days post discharge, except during charging (1–2 h per week). The Fitbit synced data frequently to the Fitbit platform. We exported the data from this platform at the end of the study. Steps and PA intensity were quantified every 24 h, starting at the time of discharge up to seven days post discharge. We omitted incomplete (zero minutes of registered PA in 24 h) days (e.g., when the participant forgot to wear the activity tracker) and days when data were not collected.

Fitbit categorizes PA into light, moderate, or vigorous intensity based on metabolic equivalents (METs) [30]. One MET is defined as the amount of oxygen consumed at rest and is equal to 3.5 ml of oxygen per kg of body weight × minutes. The Fitbit uses the estimated resting metabolic rate as a base rate to calculate the METs, however the algorithm for this calculation is not provided by Fitbit. PA with 1–3 METs is classified as light intensity (e.g., slow walking), 3–6 METs as moderate intensity (e.g., brisk walking), and > 6 METs as vigorous intensity (e.g., running). To analyze cut-off values for step numbers, we calculated the individuals’ average number of steps taken per day. For analysis of PA intensity, we used the individuals’ average minutes of PA per intensity level per day.

#### Measurement of frailty

Within 48 h after admission, we measured physical frailty using Fried’s five criteria: weight loss, low handgrip strength, low PA, slow walking speed, and fatigue [17]. Each criterion was scored as 0 (absent) or 1 (present). An individual was considered frail if three or more criteria were present. Weight loss was defined as 6 kg or more within 6 months or 3 kg or more within the past month [34]. Handgrip strength was measured three times using a dynamometer [35, 36]. The highest score from both hands was used. Low handgrip strength was defined as < 18 kg for women and < 30 kg for men [36]. Low PA was defined as fewer than 30 min of self-reported physical exercise (walking, cycling, or swimming) per month in the past 6 months before admission [17, 24]. Slow walking speed was defined as walking 4 m in more than 6.42 s [17, 27]. Fatigue was defined as a score of 4 or more in response to the question “On a scale of 0–10, how would you score your sense of fatigue at this time?” [37].

#### Measurement of recovery

Recovery was defined as the absence of functional decline, unplanned hospital readmission, and mortality at three months post discharge. Three months after hospital admission has been found to be a critical period for recovery of activities of daily living in older patients [14, 38].

Functional decline was assessed based on the participants’ ability to perform basic activities of daily living using the Katz-ADL index score [25]. Within 48 h of admission, we asked participants to rate their ability to perform ADLs during the two weeks before hospital admission. We repeated this assessment three months after discharge. We asked participants whether they needed assistance to perform each ADL and calculated a summary score ranging from 0 (independent in all ADLs) to 6 (dependent on help for all ADLs). We considered functional decline as ≥ 1 point higher dependency on help in one or more ADLs compared with two weeks before admission.

We defined an unplanned readmission as a non-elective acute admission to a hospital within three months after discharge. Data on readmissions were collected from medical files in the participating hospitals and supplemented with participants’ self-reported readmissions to other hospitals. Data on mortality during the three months after discharge were collected from medical files, family, or the general practitioner.

### Statistical analyses

We described continuous variables as a mean and standard deviation (SD) or median and interquartile range (IQR) if non-normally distributed. Categorical variables are presented as a number (n) and percentage (%). We explored the number of steps and minutes spent at different intensity levels, presented as median and IQR. We computed multivariable logistic regression models to investigate the association between PA (steps or minutes light PA) and 3-month recovery while controlling for age and gender. To improve interpretation, we used 100 steps- and 10 min PA intervals for calculations. Next, we repeated the process while controlling for additional demographic and clinical characteristics previously reported to be associated with 30-day readmission. Identified confounders were included in the final model in addition to age and gender. We analyzed the area under the curve (AUC) as a measure of accuracy for each model. An AUC of 0.5 suggests no discrimination, 0.7–0.8 is considered acceptable, 0.8–0.9 is considered excellent, and more than 0.9 is considered outstanding. We performed ROC-curve analyses (Additional file 1: figure S1) to determine cut-off values for number of steps per day and PA intensity that differentiate between recovered and non-recovered participants. Cut-off values were based on the maximized sum of sensitivity and specificity values according to the Youden index [39, 40]. ROC analyses for determining cut-offs were first performed for all participants and then separately for frail and non-frail participants. We performed a sensitivity analysis by calculating ORs for 10% higher and lower cut-offs. To check for selection bias, we compared all baseline variables between participants included in our analyses versus non-included participants. To check for the influence of outliers, we re-performed all analyses after removing outliers. All statistical analyses were performed in IBM SPSS 26.0 (IBM Corp. Released 2019. IBM SPSS Statistics for Windows, Version 26.0. Armonk, NY: IBM Corp).

## Results

**Fig. 1:**
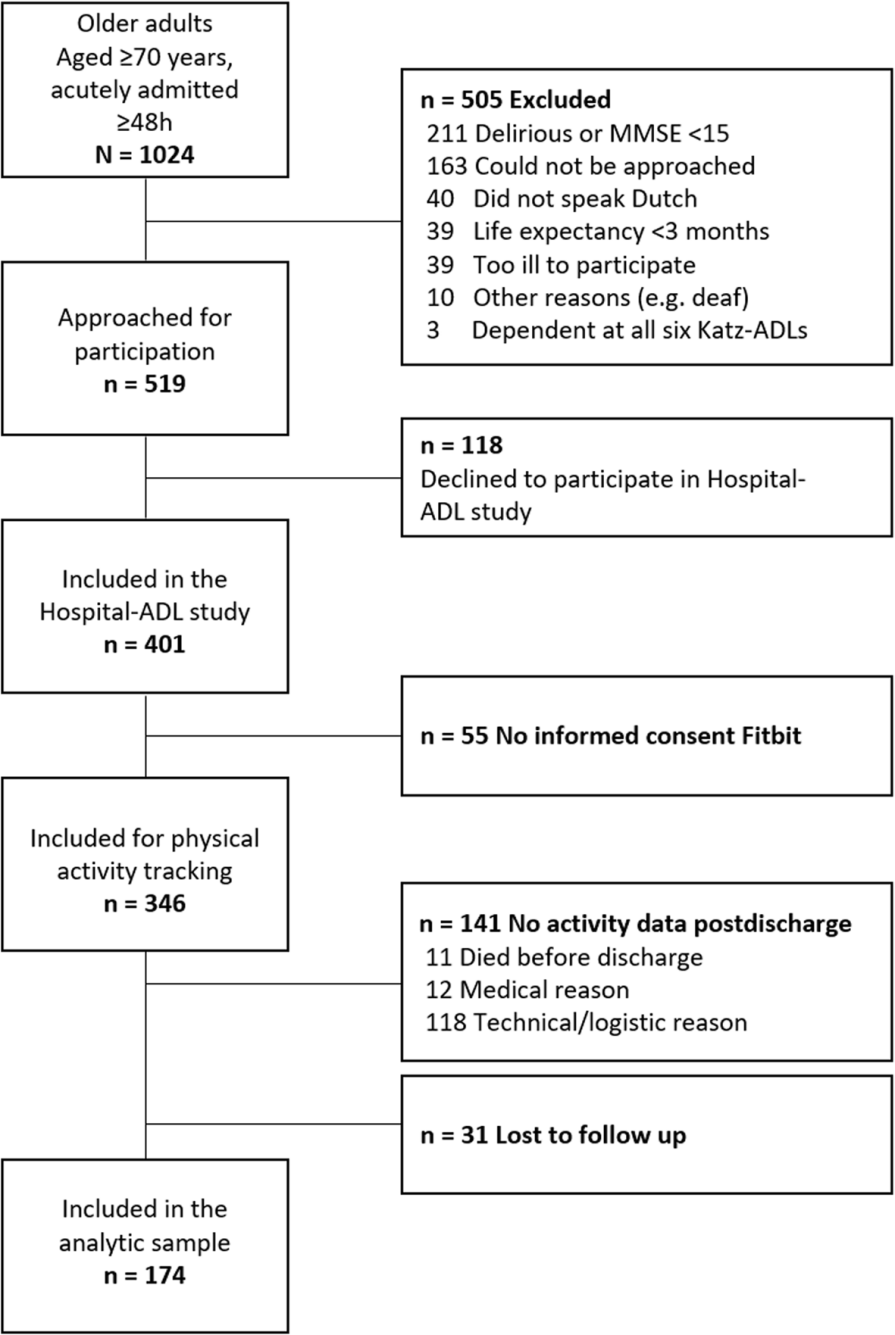
Derivation of the analytic sample. Abbreviations: MMSE, Mini-Mental State Examination; ADL, activities of daily living

Within the Hospital-ADL study (*n* = 401), 346 participants consented to wear the Fitbit. Post discharge, PA measurements were unavailable for 141 participants (Fig. 1), mostly due to technical and logistic reasons. Three months post-discharge, 31 of 205 participants with post discharge activity data (15%) were lost to follow up. The analytic sample included 174 participants who had a mean (SD) age of 79.2 (6.7) years, 91/174 (52%) were male, 156/174 (90%) were born in the Netherlands, 23 (14%) had a cognitive impairment, and 84/174 (48%) were frail (Table 1). The main primary admission diagnoses were cardiac (*n* = 57, 33%), respiratory (*n* = 29, 17%), infectional (*n* = 23, 13%), and gastrointestinal (*n* = 22, 13%) disease. Three months after discharge, 109 participants (63%) had recovered, of whom 48 were frail. Missing data analysis showed that participants not included in the analysis (*n* = 227 of *n* = 401) had a significantly lower body mass index, lower physical performance, longer hospital stay, and more frequent cognitive impairment than included participants did.

**Table 1:** Baseline Characteristics of the study population

Demographics	*N* = 174
**Age,** mean (SD), y	79.2 (6.7)
**Male,** No. (%)	91 (52)
**BMI,**^**a**^ mean (SD)	22.0 (4.1)
**Born in the Netherlands,** No. (%)	156 (90)
**Education,** No. (%)	
Primary school	37 (21)
Elementary technical/domestic science school	38 (22)
Secondary vocational education	56 (32)
Higher level high school/third-level education	43 (25)
**Primary admission diagnosis,** No. (%)	
Cardiac	57 (33)
Respiratory	29 (17)
Other	26 (15)
Infection	23 (13)
Gastrointestinal	22 (13)
Renal	6 (3)
Cancer (including hematology)	6 (3)
Electrolyte disturbance	5 (3)
**Length of hospital stay,** median (IQR), d	5 (1–9)
**Living independent after discharge,** No. (%)	134 (78)
**Clinical characteristics**	
**Charlson Comorbidity Index,**^**c**^ median (IQR)	2 (0–4)
**Polypharmacy,**^**b**^ No. (%), *(N* = *172)*	115 (67)
**Physical performance,**^**d**^ median (IQR), *(N* = *162)*	6 (0–12)
**Katz-ADL score pre-morbid,**^**e**^ median (IQR)	0 (0–1)
**FAC,** No. (%), *(N* = *163)*	
Independent	32 (20)
Independent on level surfaces	72 (44)
Dependent on supervision	35 (22)
Dependent on physical assistance I	14 (9)
Dependent on physical assistance II	3 (2)
Non-functional ambulation	7 (4)
**Cognitive impairment,**^f^ No. (%), *(N* = *169)*	23 (14)
**Frailty,**^**g**^ No, (%)	84 (48)

*SD* standard deviation, *No* number, *y* years, *d* days, *BMI* body mass index, *IQR* interquartile range, *SNAQ* short nutritional assessment questionnaire, *ADL* activities of daily living, *NRS* numeric rating scale, *FAC* functional ambulation categories

^a^Calculated as weight in kg divided by height in m^2^

^b^Use of 5 or more different medications

^c^Range of 0–31, with a higher score indicating more or severe comorbidity

^d^Assessed with the short physical performance battery. The score ranges from 0 to 12, with a higher score indicating better physical performance

^e^Ranging from 0 (independent in all ADLs) to 6 (dependent in all ADLs)

^f^Score of < 24 on the Mini-Mental State Examination

^g^Score of ≥ 2 on the Fried criteria

**Fig. 2:**
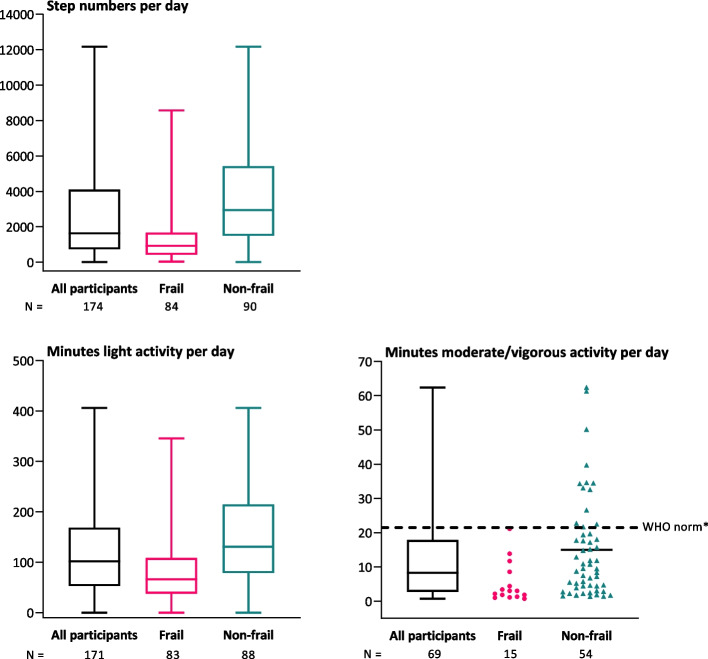
Median levels of physical activity after discharge. Horizontal lines denote median values; boxes extend from 25^th^ to the 75^th^ percentile; vertical extending lines denote the range of the number of steps and minutes of activity at light and moderate/vigorous intensity^†^ per day performed after discharge by all participants, and stratified by frail and non-frail participants. ^*^150 min of moderate intensity physical activity per week divided by seven days ≈ 21 min per day. ^†^Only participants who managed to perform this intensity are shown

Participants wore the activity tracker for a median (IQR) of 6 (5–7) days. Figure 2 shows the daily number of steps, and minutes spent performing PA at light and moderate/vigorous intensity. Participants took a median (IQR) of 1633 (735–4105) steps per day post discharge. Frail participants took a median (IQR) of 886 (421–1682) steps and non-frail participants took 3214 (1501–5767) steps.

All participants spent a median (IQR) of 102 (54–171) minutes doing light PA. Frail participants performed a median (IQR) of 79 (53–181) minutes of light PA per day, and non-frail participants performed 206 (120–250) minutes of light PA per day. Among all participants, 28/174 participants (16%) were able to do moderate/vigorous PA. Twelve participants (7%) managed more than 21 min of moderate PA per day, which is the daily level of PA recommended by the WHO (Fig. 2).

We found an optimal cut-off value of 1369 steps with an area under the curve (AUC) of 0.61 (95% CI 0.53–0.70, *p* = 0.012, sensitivity 64%, specificity 58%). In frail participants, the optimal cut-off value was 1043 steps with an AUC of 0.59 (95% CI 0.47–0.72, *p* = 0.064, sensitivity 72%, specificity 56%). In non-frail participants, the optimal cut-off value was 2611 steps with an AUC of 0.59 (95% CI 0.46–0.72, *p* = 0.168, sensitivity 61%, specificity 62%).

In all participants, we found an optimal cut-off value of 76 min of light PA with an AUC of 0.61 (95% CI 0.52–0.70, *p* = 0.022, sensitivity 70%, specificity 57%). In frail participants, the optimal cut-off was 72 min of light PA with an AUC of 0.62 (95% CI 0.50–0.74, *p* = 0.064, sensitivity 58%, specificity 75%). In non-frail older participants, the cut-off was 133 min of light PA with an AUC of 0.57 (95% CI 0.44–0.70, *p* = 0.282, sensitivity 57%, specificity 62%).

Table 2 presents the ORs for recovery three months after discharge based on the mean daily step count and minutes of light PA, and the identified optimal cut-off values for number of steps and minutes of light PA. The first models (A1, B1, AA1, BB1) are adjusted for age and gender, the second models (A2, B2, AA2, BB2) are also adjusted for the identified confounders: cognitive function, depression, admission diagnosis, and frailty. For mean daily step count and minutes of light PA, we found no association with recovery after three months. In the fully adjusted model, for the 1369-steps cut-off value we found an OR of 2.7 (95% CI 1.3–5.9) and an AUC of 0,70 (*p* = 0.00), improving the model by 8%. In frail participants (Table 3), we found an OR of 5.0 (95% CI 1.7–14.8) with an AUC of 0,72 (*p* = 0.00) for the 1043-steps cut-off value, improving the model by 11%. In non-frail participants, we found that the of 2611-steps cut-off value was not significantly associated with recovery (OR: 2.7; 95% CI 1.0–7.7). For minutes of light PA, the cut-off value of 76 min in all participants was significantly associated with recovery (OR: 3.9; 95% CI 1.8–8.5, AUC 0.73, *p* = 0.00), improving the model by 12%. In frail participants, the cut-off value of 72 min was also significantly associated with recovery (OR: 7.2; 95% CI 2.2–23.1, AUC 0.74, *p* = 0.00) and improved the model by 14%. The cut-off value of 133 min of light PA in non-frail participants was not significantly associated with recovery (OR: 2.0; 95% CI 0.7–5.5).

**Table 2:** Multivariate logistic regression model of 3-month recovery by mean daily steps (models A) and minutes light physical activity (models B) and cut-off steps (models AA) and cut-off minutes light physical activity (models BB)

		**Odds Ratio**	**95% CI**	**AUC**	***p*-value**
		Adjustment for age and gender			
A1. Step count	Per 100 steps	1.0	1.0–1.0	0.59	0.04
B1.Light activity	Per 10 min	1.0	1.0–1.1	0.59	0.04
AA1.Step count*	≥ 1369 steps	2.6	1.3–5.0	0.63	0.01
BB1.Light activity*	≥ 76 min	3.3	1.7–6.5	0.65	0.01
		Adjustment for age, gender, cognitive function, admission diagnosis, depression, frailty			
A2.Step count	Per 100 steps	1.0	1.0–1.0	0.68	0.00
B2.Light activity	Per 10 min	1.0	1.0–1.1	0.67	0.00
AA2.Step count*	≥ 1369 steps	2.7*	1.3–5.9	0.70	0.00
BB2.Light activity*	≥ 76 min	3.9*	1.8–8.5	0.73	0.00
Without steps or light activity				0.65	0.00

*CI *Confidence Interval odds ratio

^*^Statistically significant odds ratio (*p* < 0.05, CI running through 1), *p*-value = AUC statistical difference from 0.5

**Table 3:** Multivariate logistic regression model of 3-month recovery by cut-off steps and cut-off minutes light physical activity, stratified by frailty

		**Odds Ratio**	**95% CI**	**AUC**	***p*-value**
		Adjustment for age, gender, cognitive function, depression, admission diagnosis			
Frail step count^*^	≥ 1043 steps	5.0^*^	1.7–14.8	0.72	0.00
Frail light activity^*^	≥ 72 min	7.2^*^	2.2–23.1	0.74	0.00
Non-frail step count	≥ 2611 steps	2.7	1.0—7.7	0.70	0.00
Non-frail light activity	≥ 133 min	2.0	0.7—5.5	0.69	0.00
Without steps or light activity				0.65	0.00

*CI* Confidence Interval odds ratio

^*^Statistically significant odds ratio (CI running through 1), *p*-value = AUC statistical difference from 0.5

The sensitivity analyses showed that slightly different cut-off values gave lower ORs which were in the same direction and remained significant among all participants and in the frail group. In the non-frail group, a lower steps cut-off of 2350 steps gave a higher OR and changed to be statistically significant (OR: 2.5; 95% CI 1.0 – 6.3). Removing outliers did not influence the outcomes of the analyses.

## Discussion

We found that performing more than 1369 steps and 76 min of light physical activity per day in the first week after hospital discharge differentiates older adults who were recovered at three months post discharge. We focused on light PA because we found that not all older adults were able to do moderate- or high-intensity PA. In frail older adults, we identified a cut-off value of 1043 steps and 72 min of light PA per day. The cut-off values in non-frail older adults were higher and not significantly associated with recovery.

Few participants were able to meet the PA levels currently recommended by the WHO [1]. The WHO recommends 150 min of moderate to intense PA per week; to achieve this, an individual would have to take at least 7000–8000 steps per day [10]. Tudor-Locke et al. have already suggested that recommended PA levels should be lowered for special populations but offered no concrete recommendations. While many studies have investigated step counts in hospitalized older adults [5, 12, 18, 41–44], ours is the first to recommend cut-off values for post-discharge step count and minutes of PA at specific intensities per day. The cut-off value of 1369 steps identified by us is higher than the cut-off value of 900 steps in the hospital from previous research [18]. This difference can be explained by the fact that older people were more active in the first week after discharge than during admission [12]. An important addition of our study is that we also investigated cut-offs for intensity of PA. This might be a more representative measure of PA since it includes more activities, like cycling or household tasks. Especially in older adults, step counts may underestimate the intensity of PA, particularly at low walking speeds (< 3 mph) [45].

Cut-off values for PA can help clinicians predict the chance of recovery in older adults following acute hospitalization. The cut-off values and the use of wrist worn activity trackers can also provide patients insight into their recovery process and may encourage them to become more active and engaged [46]. Number of steps and intensity of PA are easy to measure using wearable technology without the need for a health professional, which is an important advantage in the care for older adults after being discharged. Moreover, activity trackers, can measure PA over a longer period, which may give more realistic outcomes than point estimates given by for example, a physical performance, strength or walking speed test [22, 27, 47].

In line with a recent study in the general population, our findings show that light-intensity PA is sufficient for recovery in frail older adults after hospital discharge [48]. Our results also show that especially in frail older adults, PA levels might be a simple physical function indicator of recovery. We found that PA levels were higher in non-frail older adults, but AUCs were lower, and cut-off values were not significantly associated with recovery, showing limited potential for application of the cut-off in non-frail. This might be explained by the model of Fried et al. [17], suggesting that frailty is associated with more decline in physical health in response to acute illness. Therefore, PA levels might be more reduced in frail older adults and may better reflect their potential to recover. These findings also highlight the importance to identify frailty in acutely hospitalized older adults.

### Strengths and limitations

This study has several potential limitations. We included participants with a wide variety of primary admission diagnoses and 23% of the participants were cognitively impaired because this is representative for the geriatric population. Although we corrected for these factors in the multivariate analyses, the optimal amount of PA can differ per individual. We excluded participants with dependency on all basic ADLs. Also, many participants did not wear the activity tracker after discharge which may lead to an underestimation of the observed associations and a reduced generalizability of our findings. The Fitbit Flex needs to be worn to measure PA; therefore, PA may have been underestimated if the participant was not wearing the tracker. The Fitbit Flex uses a standard non-disclosed algorithm, based on a standard length and weight, and on healthy adults, to calculate energy expenditure, which is then used to assess the level of PA. Therefore, the algorithm may misjudge the actual energy expenditure. Both under- and overestimation of energy expenditure by Fitbit has been reported [31, 49]. Because of this limited accuracy, our reported cut-offs for minutes at PA intensity levels should by applied with caution, although the low levels of PA seem to be appropriate for our population. An acceptable reliability and high validity for counting steps has been reported for Fitbit [31, 50]. However, an underestimation of steps has been reported in older adults, especially when using a walker [33]. It is therefore wise to check the reliability of pedometers on an individual basis and to be cautious in generalizing our results to other wearables.

We used a composite recovery outcome, which may differ substantially between individuals. This limits conclusions about specific outcomes. However, using a composite outcome increased the power of our study and is congruent with other cut-off studies [18, 19].

Another limitation of this study is that the AUCs were moderate in our study. This shows that post discharge PA measurement is not an ideal test for determining who is at risk of non-recovery. However, we also wanted to investigate the association between these cut-off values and recovery. This has been investigated by Agmon et al. during hospitalization [18], but not yet after hospitalization. Therefore, we investigated the performance of the optimal cut-off points by analysing the odds ratios for recovery. Less robust cut-offs were found in the non-frail group, suggesting that the association of PA and recovery is less strong among non-frail older adults. However, among all participants and frail participants, the cut-off values were robust and gave the best odds ratios. Nevertheless, these cut-offs should not be used as strict norms, but as a guidance in clinical practice.

## Conclusions and implications

Our study is the first to describe the association between post-discharge PA levels and recovery in older patients after hospital discharge. Many acutely hospitalized older adults did not achieve moderate or vigorous PA post discharge, which in the first week after discharge did not seem to be essential for recovery. Considering the low AUC’s, the identified cut-offs are not equipped for use as a diagnostic test in daily practice, however they can be seen as a first step to provide a direction for setting rehabilitation goals. A clinical trial is necessary to evaluate if PA goals above the cut-off will improve recovery, especially in frail older adults.

## Supplementary Information

**Additional file 1.** Receiver operating curves for determining cut-off values.

## Data Availability

The datasets generated and/or analysed during the current study are not publicly available due to privacy, but are available from the corresponding author on reasonable request.

## References

[1] World Health Organization (2011). Global recommendations on physical activity for health 65 years and above.

[2] DiPietro L, Buchner DM, Marquez DX, Pate RR, Pescatello LS, Whitt-Glover MC (2019). New scientific basis for the 2018 U.S. physical activity guidelines. J Sport Health Sci..

[3] Whitson HE, Cohen HJ, Schmader KE, Morey MC, Kuchel G, Colon-Emeric CS (2018). Physical Resilience: Not Simply the Opposite of Frailty. J Am Geriatr Soc.

[4] Ewald B, Attia J, McElduff P (2014). How many steps are enough? Dose-response curves for pedometer steps and multiple health markers in a community-based sample of older Australians. J Phys Act Health.

[5] Fisher SR, Graham JE, Ottenbacher KJ, Deer R, Ostir GV (2016). Inpatient walking activity to predict readmission in older adults. Arch Phys Med Rehabil.

[6] Beagle AJ, Tison GH, Aschbacher K, Olgin JE, Marcus GM, Pletcher MJ (2020). Comparison of the Physical Activity Measured by a Consumer Wearable Activity Tracker and That Measured by Self-Report: Cross-Sectional Analysis of the Health eHeart Study. JMIR Mhealth Uhealth.

[7] VandeBunte A, Gontrum E, Goldberger L, Fonseca C, Djukic N, You M (2022). Physical activity measurement in older adults: Wearables versus self-report. Front Digit Health.

[8] World Health Organization (2010). Global recommendations on physical activity for health.

[9] Health USDo, Human S. Physical Activity Guidelines for Americans: Be Active, Healthy, and Happy!. 2008. https://www.healthgov/paguidelines/guidelines/defaultaspx.

[10] Tudor-Locke C, Craig CL, Aoyagi Y, Bell RC, Croteau KA, De Bourdeaudhuij I (2011). How many steps/day are enough? For older adults and special populations. Int J Behav Nutr Phys Act.

[11] Titze S, Lackinger C, Fessl C, Dorner TE, Zeuschner V (2020). Austrian physical activity guidelines for adults and older adults with and without physical, sensory, or mental disabilities, as well as for adults with chronic diseases. Gesundheitswesen.

[12] Kolk D, Aarden JJ, MacNeil-Vroomen JL, Reichardt LA, van Seben R, van der Schaaf M (2020). Factors associated with step numbers in acutely hospitalized older adults: the hospital-activities of daily living study. J Am Med Dir Assoc.

[13] Buurman BM, van den Berg W, Korevaar JC, Milisen K, de Haan RJ, de Rooij SE (2011). Risk for poor outcomes in older patients discharged from an emergency department: feasibility of four screening instruments. Eur J Emerg Med.

[14] Boyd CM, Landefeld CS, Counsell SR, Palmer RM, Fortinsky RH, Kresevic D (2008). Recovery of activities of daily living in older adults after hospitalization for acute medical illness. J Am Geriatr Soc.

[15] Cunha AIL, Veronese N, de Melo Borges S, Ricci NA (2019). Frailty as a predictor of adverse outcomes in hospitalized older adults: A systematic review and meta-analysis. Ageing Res Rev.

[16] Wou F, Gladman JR, Bradshaw L, Franklin M, Edmans J, Conroy SP (2013). The predictive properties of frailty-rating scales in the acute medical unit. Age Ageing.

[17] Fried LP, Tangen CM, Walston J, Newman AB, Hirsch C, Gottdiener J (2001). Frailty in older adults: evidence for a phenotype. J Gerontol A Biol Sci Med Sci.

[18] Agmon M, Zisberg A, Gil E, Rand D, Gur-Yaish N, Azriel M (2017). Association between 900 steps a day and functional decline in older hospitalized patients. JAMA Intern Med.

[19] Fisher SR, Kuo YF, Sharma G, Raji MA, Kumar A, Goodwin JS (2013). Mobility after hospital discharge as a marker for 30-day readmission. J Gerontol A Biol Sci Med Sci.

[20] Fisher SR, Kuo YF, Graham JE, Ottenbacher KJ, Ostir GV (2010). Early ambulation and length of stay in older adults hospitalized for acute illness. Arch Intern Med.

[21] Brown CJ, Friedkin RJ, Inouye SK (2004). Prevalence and outcomes of low mobility in hospitalized older patients. J Am Geriatr Soc.

[22] Volpato S, Cavalieri M, Sioulis F, Guerra G, Maraldi C, Zuliani G (2011). Predictive value of the short physical performance battery following hospitalization in older patients. J Gerontol A Biol Sci Med Sci.

[23] Suboc TB, Strath SJ, Dharmashankar K, Coulliard A, Miller N, Wang J (2014). Relative importance of step count, intensity, and duration on physical activity's impact on vascular structure and function in previously sedentary older adults. J Am Heart Assoc.

[24] Reichardt LA, Aarden JJ, van Seben R, van der Schaaf M, Engelbert RH, Bosch JA (2016). Unravelling the potential mechanisms behind hospitalization-associated disability in older patients; the Hospital-Associated Disability and impact on daily Life (Hospital-ADL) cohort study protocol. BMC Geriatr.

[25] Katz S, Downs TD, Cash HR, Grotz RC (1970). Progress in development of the index of ADL. Gerontologist.

[26] Charlson ME, Pompei P, Ales KL, MacKenzie CR (1987). A new method of classifying prognostic comorbidity in longitudinal studies: development and validation. J Chronic Dis.

[27] Guralnik JM, Simonsick EM, Ferrucci L, Glynn RJ, Berkman LF, Blazer DG (1994). A short physical performance battery assessing lower extremity function: association with self-reported disability and prediction of mortality and nursing home admission. J Gerontol.

[28] Holden MK, Gill KM, Magliozzi MR, Nathan J, Piehl-Baker L (1984). Clinical gait assessment in the neurologically impaired Reliability and meaningfulness. Phys Ther.

[29] Folstein MF, Folstein SE, McHugh PR (1975). "Mini-mental state". A practical method for grading the cognitive state of patients for the clinician. J Psychiatr Res.

[30] Brewer W, Swanson BT, Ortiz A (2017). Validity of Fitbit's active minutes as compared with a research-grade accelerometer and self-reported measures. BMJ Open Sport Exerc Med.

[31] Evenson KR, Goto MM, Furberg RD (2015). Systematic review of the validity and reliability of consumer-wearable activity trackers. Int J Behav Nutr Phys Act.

[32] Straiton N, Alharbi M, Bauman A, Neubeck L, Gullick J, Bhindi R (2018). The validity and reliability of consumer-grade activity trackers in older, community-dwelling adults: a systematic review. Maturitas.

[33] Burton E, Hill KD, Lautenschlager NT, Thogersen-Ntoumani C, Lewin G, Boyle E (2018). Reliability and validity of two fitness tracker devices in the laboratory and home environment for older community-dwelling people. BMC Geriatr.

[34] Kruizenga HM, Seidell JC, de Vet HC, Wierdsma NJ, van Bokhorst-de van der Schueren MA (2005). Development and validation of a hospital screening tool for malnutrition: the short nutritional assessment questionnaire (SNAQ). Clin Nutr..

[35] Trutschnigg B, Kilgour RD, Reinglas J, Rosenthall L, Hornby L, Morais JA (2008). Precision and reliability of strength (Jamar vs. Biodex handgrip) and body composition (dual-energy X-ray absorptiometry vs. bioimpedance analysis) measurements in advanced cancer patients. Appl Physiol Nutr Metab.

[36] Roberts HC, Denison HJ, Martin HJ, Patel HP, Syddall H, Cooper C (2011). A review of the measurement of grip strength in clinical and epidemiological studies: towards a standardised approach. Age Ageing.

[37] Hwang SS, Chang VT, Cogswell J, Kasimis BS (2002). Clinical relevance of fatigue levels in cancer patients at a Veterans Administration Medical Center. Cancer.

[38] Buurman BM, Hoogerduijn JG, de Haan RJ, Abu-Hanna A, Lagaay AM, Verhaar HJ (2011). Geriatric conditions in acutely hospitalized older patients: prevalence and one-year survival and functional decline. PLoS One.

[39] Liu X (2012). Classification accuracy and cut point selection. Stat Med.

[40] Hajian-Tilaki K (2018). The choice of methods in determining the optimal cut-off value for quantitative diagnostic test evaluation. Stat Methods Med Res.

[41] Arentson-Lantz E, Galvan E, Wacher A, Fry CS, Paddon-Jones D (2019). 2,000 steps/day does not fully protect skeletal muscle health in older adults during bed rest. J Aging Phys Act.

[42] Cook DJ, Thompson JE, Prinsen SK, Dearani JA, Deschamps C (2013). Functional recovery in the elderly after major surgery: assessment of mobility recovery using wireless technology. Ann Thorac Surg.

[43] Lee IM, Shiroma EJ, Kamada M, Bassett DR, Matthews CE, Buring JE (2019). Association of Step Volume and Intensity With All-Cause Mortality in Older Women. JAMA Intern Med.

[44] Sallis R, Roddy-Sturm Y, Chijioke E, Litman K, Kanter MH, Huang BZ (2015). Stepping toward discharge: level of ambulation in hospitalized patients. J Hosp Med.

[45] Bassett DR, Toth LP, LaMunion SR, Crouter SE (2017). Step counting: a review of measurement considerations and health-related applications. Sports Med.

[46] Pol M, Peek S, van Nes F, van Hartingsveldt M, Buurman B, Krose B (2019). Everyday life after a hip fracture: what community-living older adults perceive as most beneficial for their recovery. Age Ageing.

[47] Marzetti E, Calvani R, Tosato M, Cesari M, Di Bari M, Cherubini A (2017). Physical activity and exercise as countermeasures to physical frailty and sarcopenia. Aging Clin Exp Res.

[48] Saint-Maurice PF, Troiano RP, Bassett DR, Graubard BI, Carlson SA, Shiroma EJ (2020). Association of daily step count and step intensity with mortality among US Adults. JAMA.

[49] Feehan LM, Geldman J, Sayre EC, Park C, Ezzat AM, Yoo JY (2018). Accuracy of Fitbit Devices: Systematic Review and Narrative Syntheses of Quantitative Data. JMIR Mhealth Uhealth.

[50] Alharbi M, Bauman A, Neubeck L, Gallagher R (2016). Validation of Fitbit-Flex as a measure of free-living physical activity in a community-based phase III cardiac rehabilitation population. Eur J Prev Cardiol.

