# Supplementary material for: Post-discharge light physical activity indicates recovery in acutely hospitalized older adults – the Hospital-ADL study
Source: BMC Geriatr. 2023 May 19;23:311. doi: 10.1186/s12877-023-04031-9 (PMC10197221; doi:10.1186/s12877-023-04031-9)
Supplement: Supplementary file 1 — Additional file 1. Receiver operating curves for determining cut-off values. [file 12877_2023_4031_MOESM1_ESM.docx]

Additional file 1. Receiver operating curves for determining cut-off values


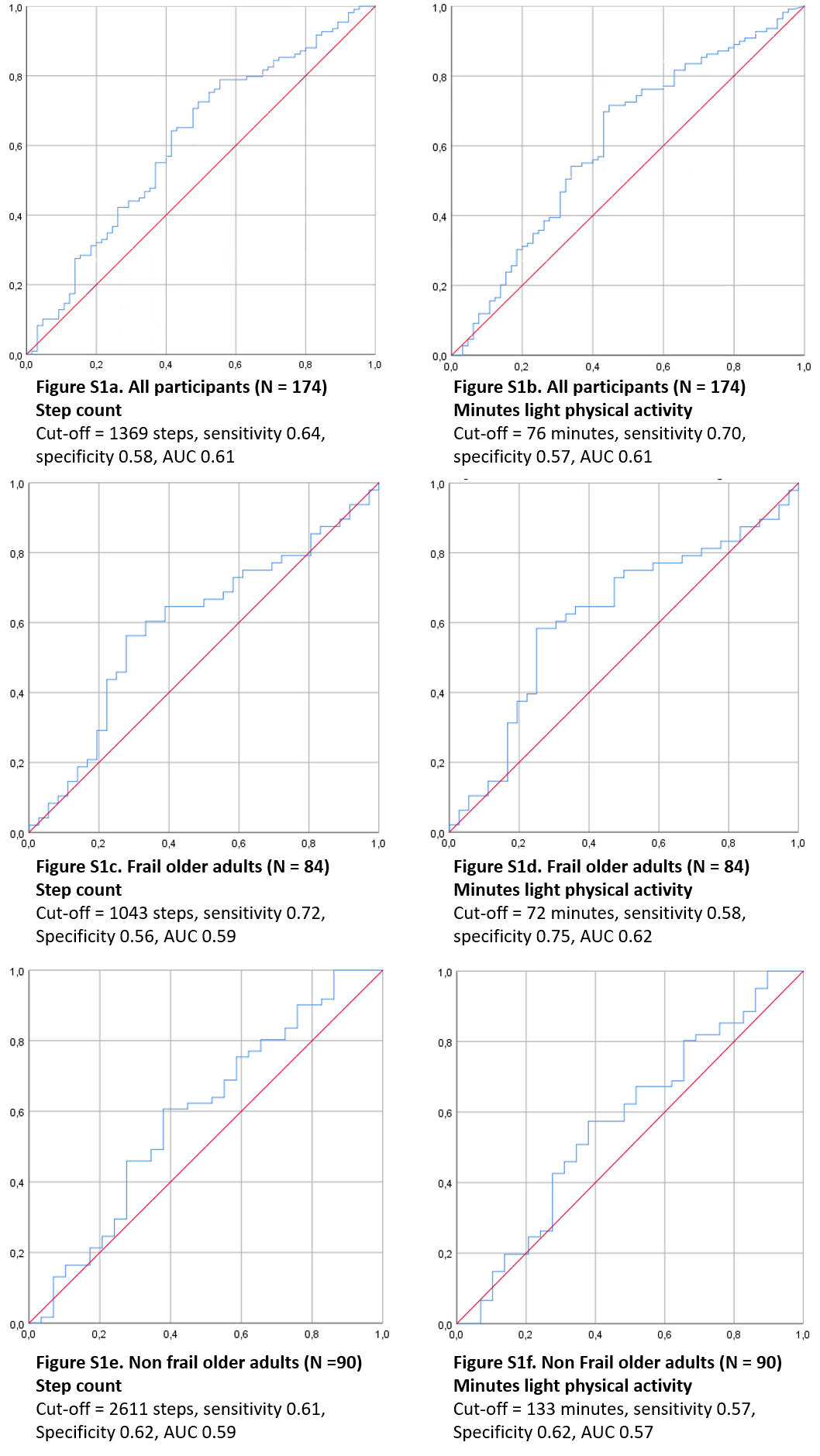


**Figure S1. Receiver operating curves of older adults recovery 3 months**

**post discharge based on levels of physical activity measured in steps**

**or minutes of light activity.** The x-axis displays 1 – specificity (false

positive rate), the y-axis displays the sensitivity (true positive rate),

AUC = area under the curve.
